# Three Melanin Pathway Genes, *TH*, *yellow*, and *aaNAT*, Regulate Pigmentation in the Twin-Spotted Assassin Bug, *Platymeris biguttatus* (Linnaeus)

**DOI:** 10.3390/ijms20112728

**Published:** 2019-06-03

**Authors:** Yinqiao Zhang, Hu Li, Juan Du, Junzheng Zhang, Jie Shen, Wanzhi Cai

**Affiliations:** Department of Entomology and MOA Key Lab of Pest Monitoring and Green Management, College of Plant Protection, China Agricultural University, Beijing 100193, China; zhangyinqiao519@126.com (Y.Z.); tigerleecau@hotmail.com (H.L.); dujuan9981@163.com (J.D.); 2015008@cau.edu.cn (J.Z.)

**Keywords:** *Platymeris biguttatus*, pigmentation, tyrosine hydroxylase (*TH*), *yellow*, arylalkylamine-*N*-acetyltransferase (*aaNAT*)

## Abstract

Pigmentation plays a vital role in insect survival and reproduction. Many melanin pathway genes have been studied in holometabolous insects; however, they have only been studied in two hemimetabolous insect genera, *Oncopeltus* and *Periplaneta*. Here we analyzed three melanin pathway genes (*TH*, *yellow*, and *aaNAT*) using RNA interference (RNAi) in another hemimetabolous insect, namely the twin-spotted assassin bug, *Platymeris biguttatus*. *TH* was highly expressed in freshly molted nymphs and adults. *TH* RNAi resulted in a complete loss of black pigment, with yellow coloration maintained. Therefore, black pigment in this assassin bug is solely generated from the melanin pathway, whereas yellow pigment is generated from other unknown pigmentation pathways. *yellow* and *aaNAT* were highly expressed in the white spot of the hemelytra. Downregulation of *yellow* caused a brown phenotype with high mortality, indicating an important role of *yellow* functions in cuticle formation and in the process of converting melanin from brown to black. Interestingly, *aaNAT* RNAi caused not only loss of white pigment, but also loss of yellow and red pigments. This phenotype of *aaNAT* has not been reported in other insects. Our results provide new information for understanding the melanin pathway in which *aaNAT* is essential for the formation of colorless patterns.

## 1. Introduction

Pigmentation is one of the most distinctive and variable features of insects. Pigmentation differs substantially between insect species, populations, and individuals, and even in different regions of the body [1,2]. Pigmentation also plays indispensable roles in behavioral, physiological, and reproductive processes [3]. Most genetic and molecular mechanisms controlling pigmentation have been elucidated in the model species *Drosophila melanogaster* [4,5,6]. Pigment synthesis begins with the conversion of tyrosine to dihydroxyphenylalanine (DOPA) by tyrosine hydroxylase, which is encoded by the *TH* gene. Some DOPA molecules are then converted to DOPA melanin (black), and the *yellow*-family genes play important roles in this process. DOPA can also be converted to dopamine by DOPA decarboxylase (Ddc), after which dopamine can go through three metabolic branches. The first branch involves the direct production of dopamine melanin (brown/black). The second branch involves conversion to *N*-β-alanyl dopamine (NBAD) by *ebony*, which generates yellow pigment, and can be reversed by *tan*. The last branch involves the production of N-acetyl dopamine (NADA), which is the precursor of colorless sclerotin, and this process depends on the activity of arylalkylamine-*N*-acetyltransferase (*aaNAT*). These melanin precursors are synthesized and secreted from the imaginal epidermis and then incorporated into the cuticle [7,8,9].

Functional analyses in *Drosophila* first shed light on basic information about genes that regulate pigmentation, such as *Ddc*, *yellow*, *tan*, and *ebony* [6,8,10,11,12]. Pigmentation has also been studied in other groups of insects. For example, *Tribolium castaneum* serves as another influential insect model, and its melanin genes (*TH*, *Ddc*, *yellow*, and *ebony*) have been studied [13,14,15,16,17]. In addition, pigmentation studies have been conducted in some other holometabolous insects, such as in *Heliconius*, *Papilio*, *Monochamus*, *Apis*, and *Aedes* genera [18,19,20,21,22,23,24]. Since 2010, the role of *aaNAT* in pigmentation was functionally analyzed in *Bombyx mori*, and it was found that RNAi against *aaNAT* caused a partial or complete black phenotype in adults [25,26,27]. In *Tribolium*, *aaNAT* was not required for pigmentation of non-adult cuticles, although it played critical roles in the morphology and pigmentation of adult cuticles [28]. To date, systematic research of these melanin genes has been performed only in two hemimetabolous insects, *Oncopeltus* and *Periplaneta* [29,30,31]. RNAi against *aaNAT* in these two hemimetabolous insects caused region-specific phenotypes. In *Oncopeltus*, only the anal lobe region of the hindwing became melanized in *aaNAT* RNAi adults. In *Periplaneta*, only the colorless region of the pronotum became melanized in ds*aaNAT*-treated adults [30]. Therefore, the genes involved in pigmentation are functionally diverse in these two types of metamorphosis in insects. Naturally, questions arise, such as whether all melanin genes work differently in holometabolous and hemimetabolous insects, or whether melanin genes function similarly among all hemimetabolous insects. Therefore, it is necessary to perform functional analyses of the melanin pathway in other hemimetabolous insects.

*Platymeris biguttatus*, with brightly contrasting colors, is very suitable for performing pigmentation studies. It is mostly black throughout the body with a yellow annulation on the leg and a white spot on the hemelytra. In addition, this insect has a body length of approximately 32 mm, and shows high efficiency of double-stranded RNA (dsRNA) injection. The expression profiles of three essential genes in the melanin pathway, *TH*, *yellow*, and *aaNAT*, were analyzed along with developmental stages, pigmentation time points, and specific tissues with respect to color patterns. The functions of these genes were assayed by RNAi. Our results suggest that the black pigmentation was solely generated from the melanin pathway in this assassin bug. In addition, downregulation of *yellow* caused a brown phenotype with high mortality. Interestingly, *aaNAT* RNAi induced completely black pigmentation, with the loss of all other color patterns.

## 2. Results

### 2.1. Expression Patterns of Three Melanin Pathway Genes (TH, yellow, and aaNAT)

**Figure 1:**
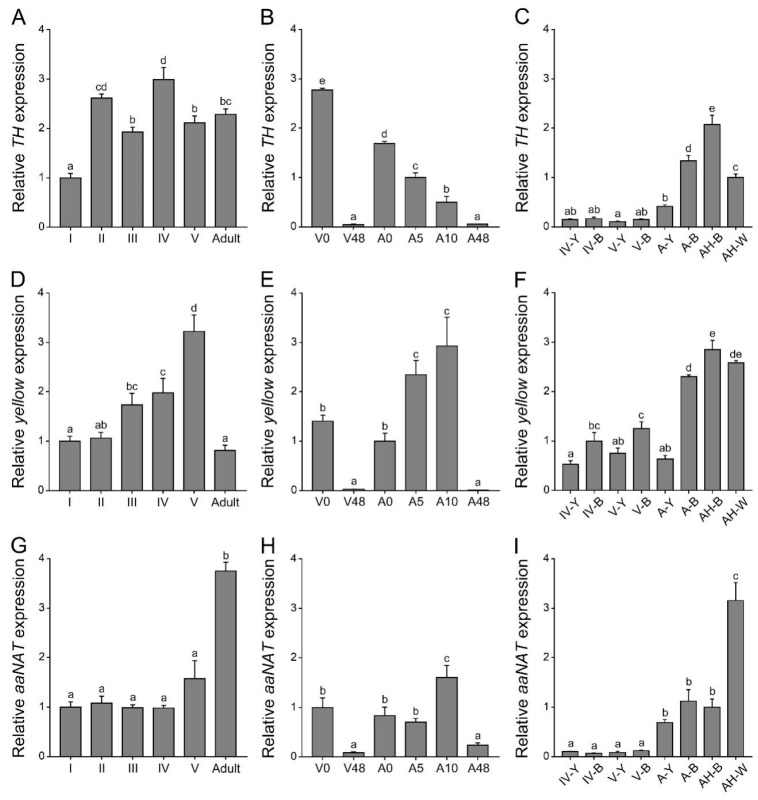
Expression profiles of *TH, yellow*, and *aaNAT* in *P. biguttatus*. (**A**,**D**,**G**) Relative expression levels of *TH*, *yellow*, and *aaNAT* were detected by quantitative real-time PCR at different developmental stages. All these nymphs and adults have completed the pigmentation process before RNA extraction. (**B**,**E**,**H**) Relative expression levels of these three genes at different pigmentation time points. (**C**,**F**,**I**) Relative expression levels of these three genes in body regions with different color patterns from different developmental stages. I–V, the first- to fifth-instar nymphs; A, adults; 0, 5, 10, and 48 h of the corresponding instar nymphs; Y, the yellow annulation of the leg; B, the black region of the leg; H-B, the black region of the hemelytra; H-W, the white spot of the hemelytra. Expression levels were normalized to the expression of *EF1α* of *P. biguttatus*. Data are shown as the mean values ± SE (error bars) (*n* = 3). Different letters (a, b, c, d, and e) on the error bars indicate statistically significant differences (one-way ANOVA analysis, *p* < 0.05).

Before using the RNAi method to analyze the functions of *TH*, *yellow*, and *aaNAT* in the color formation of this assassin bug, it was necessary to determine their expression patterns. We performed quantitative real-time PCR of these three genes at different developmental stages, different pigmentation time points, and body regions with different colors (Figure 1). The expression level of *TH* at different developmental stages was fluctuant. It was significantly lower in the first-instar nymphs than in others (Figure 1A). During the pigmentation process, the highest expression level of *TH* was detected in the freshly molted fifth-instar nymphs or adults, and gradually decreased until the pigmentation was completed (Figure 1B). *TH* expression level was significantly higher in the black and white regions of adults than in other regions (Figure 1C). The expression level of *yellow* gradually increased from the first- to the fifth-instar nymphs, but significantly declined in adults (Figure 1D). In contrast, the expression level of *aaNAT* in adults was significantly higher than that in nymphs (Figure 1G). Similar to *TH*, expression levels of *yellow* and *aaNAT* were significantly higher in the 0 h nymphs or adults than those in the 48 h nymphs or adults (Figure 1E,H). During the pigmentation process, the expression level of *yellow* was significantly increased in the 5 h adults and 10 h adults (Figure 1E), while the expression level of *aaNAT* was the highest in the 10 h adults (Figure 1H). Interestingly, *yellow* expression level in the white spot of the hemelytra was as high as in the black regions of adults (Figure 1F). The expression level of *aaNAT* was significantly higher in the white spot than in other regions (Figure 1I).

Significant differences in the temporal and spatial expression profiles were noted among these three genes. RNAi experiments are needed to determine what roles they play during the pigmentation process in this assassin bug and what, if any, relationships occur between them.

### 2.2. Functional Analyses of TH and yellow

The freshly molted fifth-instar nymph or adult was bright red, gradually turned brown after about five hours (Figures S1B and S2B), then turned dark brown after about ten hours (Figure S2C), and finally turned black after the pigmentation progress was completed (Figures S1C and S2D). This process took 18 h and 48 h for the fifth-instar nymph and the adult, respectively. The fully pigmented fifth-instar nymph was mostly black, with a yellow annulation on the leg (Figure S1C). The abdomen changed from black to brownish red after feeding (Figure S1D). Similarly, the body of the adult was mostly black, with a yellow annulation on the leg and a white spot on the hemelytra (Figure S2D). The region of the dorsal abdomen covered by the forewings was red (Figure S2E). The membranous hindwing was light brown with a fenestrate area (Figure S2F, the black arrowhead). To explore the role of the melanin pathway in the pigmentation of *P. biguttatus*, we selected three essential melanin genes: *TH*, *yellow*, and *aaNAT* to perform RNAi experiments based on the color pattern of this assassin bug. The phylogenetic analysis confirmed that *TH*, *yellow*, and *aaNAT* represent orthologs of this assassin bug (Figure S3). Then, we performed quantitative real-time PCR analysis on each gene to test the efficiency of the RNAi experiments, and the results showed that the transcription levels of these genes were significantly lower in the dsRNA-treated groups than in the ds*GFP*-treated groups (Figure S4). Therefore, the observed phenotype was caused by the decrease in the expression level of the corresponding gene. 

**Figure 2:**
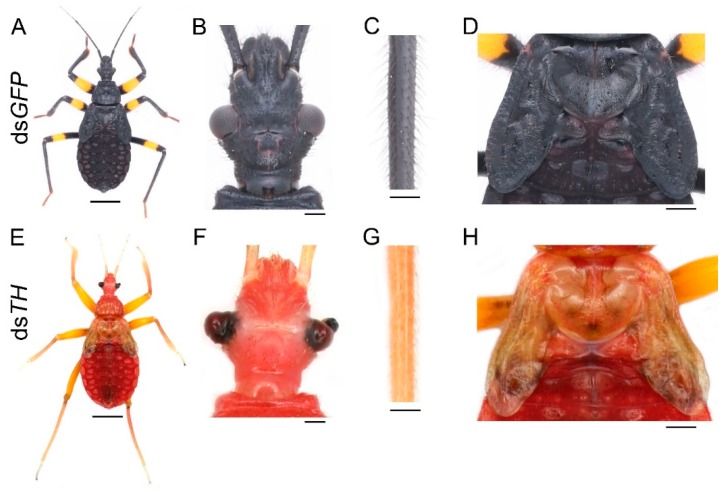
Functions of *TH* in *P. biguttatus* nymphs. Nymphs treated with ds*GFP* showed normal pigmentation patterns (**A**), well-developed cuticles (**B**,**D**), and bristles (**C**). The ds*TH* nymphs displayed nearly a complete loss of black pigment, with yellow pigment not affected (**E**). The deformed compound eyes and white bristles on the head (**F**). The short and limp bristles on the tibia (**G**). The wrinkled wing discs and some dark brown markings on the cuticle (**H**). Scale bars, 5 mm (**A**,**E**), 500 μm (**B**,**F**), 250 μm (**C**,**G**), 1.25 mm (**D**,**H**).

First, we needed to determine whether the black pigment of this species was synthesized by the melanin pathway. Since *TH* catalyzes the initial step of this pathway, we injected 20 μg ds*TH* into each of the early fourth-instar nymphs (about seven days old). The same amount of ds*GFP* was injected into the nymph of the same developmental stage as a control. In total, of the 11 ds*GFP* nymphs, three were used to extract RNA, seven molted into the fifth instar, and one died 15 days after the injection (in the middle instar). Nymphs treated with ds*GFP* molted into the fifth instar with a normal pigmentation pattern (Figure 2A). Of the 19 nymphs injected with ds*TH*, three were used to extract RNA, only four of them successfully molted to the next instar, and 12 died during the molting process. The four fifth-instar nymphs also died within seven days and were disabled in feeding. RNAi of *TH* led to the loss of black pigment throughout the body, but yellow pigment was not affected (Figure 2E), and the effects were consistent among these four fifth-instar nymphs. Some bristles were light brown or even white, especially on the head and tibia of the ds*TH* nymphs (Figure 2F,G). However, there were still some dark brown markings on the cuticle (Figure 2E,H). Down regulation of *TH* also caused defects in cuticle sclerotization. This resulted in warped legs (Figure 2E), deformed compound eyes (Figure 2F), and wrinkled wing discs (Figure 2H). The bristles of ds*GFP*-treated nymphs were long and straight (Figure 2B,C), but a lot of the bristles of the ds*TH*-treated nymphs were short and limp (Figure 2F,G). Moreover, the cuticle of the ds*TH* nymphs was less granulose and smoother than that of the control nymphs.

**Figure 3:**
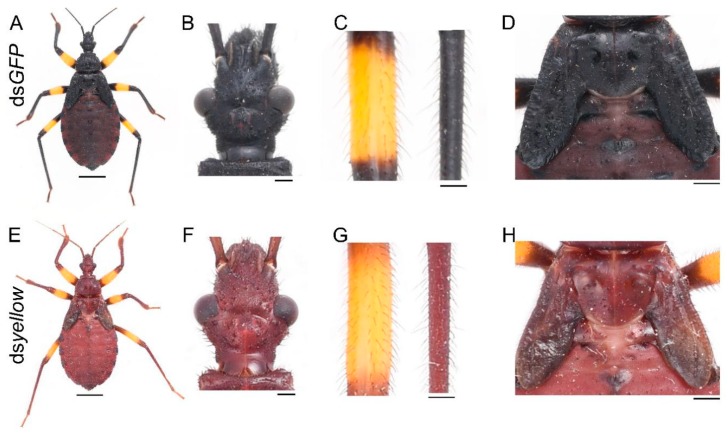
Functions of *yellow* in *P. biguttatus* nymphs. Nymphs treated with ds*GFP* showed normal pigmentation patterns (**A**), well-developed cuticles (**B**,**D**) and bristles (**C**). Nymphs injected with ds*yellow* showed a brown phenotype (**E**), with intact cuticles (**F**,**H**) and bristles (**G**). Scale bars, 5 mm (**A**,**E**), 500 μm (**B**,**F**), 250 μm (**C**,**G**), 1.25 mm (**D**,**H**).

The *yellow* gene can promote the cuticular melanization process. To determine its function in this assassin bug, we performed *yellow* RNAi experiments. Of the 21 nymphs injected with ds*yellow*, three were used to extract RNA, 16 of them died as fourth-instar nymphs during the expected molting period, and their body walls were very soft before they died, only two successfully molted to fifth-instar nymphs, and they died in the early instar. These two *yellow* RNAi nymphs showed a brown phenotype, with the lack of black pigment (Figure 3E). Loss of *yellow* function did not affect the normal development of cuticles (Figure 3F,H) or bristles (Figure 3G).

### 2.3. Functional Analyses of aaNAT

**Figure 4:**
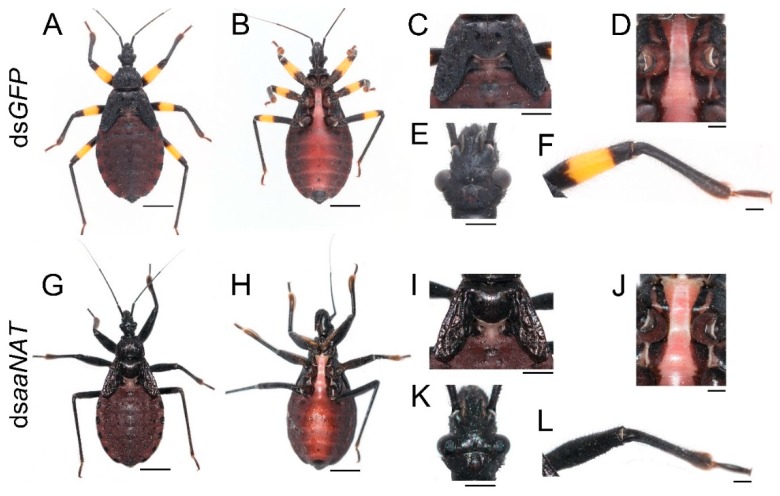
Functions of *aaNAT* in *P. biguttatus* nymphs. Nymphs treated with ds*GFP* showed normal pigmentation patterns of the dorsal (**A**) and ventral side (**B**), including the wing discs (**C**), the sternum (**D**), the head (**E**), and the leg (**F**). Nymphs treated with ds*aaNAT* showed darker pigmentation patterns than those treated with ds*GFP* (**G**–**I**,**K**), the yellow annulation of the leg turned black (**L**), while the color of sternum was not apparently changed (**J**). Scale bars, 5 mm (**A**,**B**,**G**,**H**), 2.5 mm (**C**,**I**), 1.25 mm (**E**,**K**), 1 mm (**D**,**F**,**J**,**L**).

**Figure 5:**
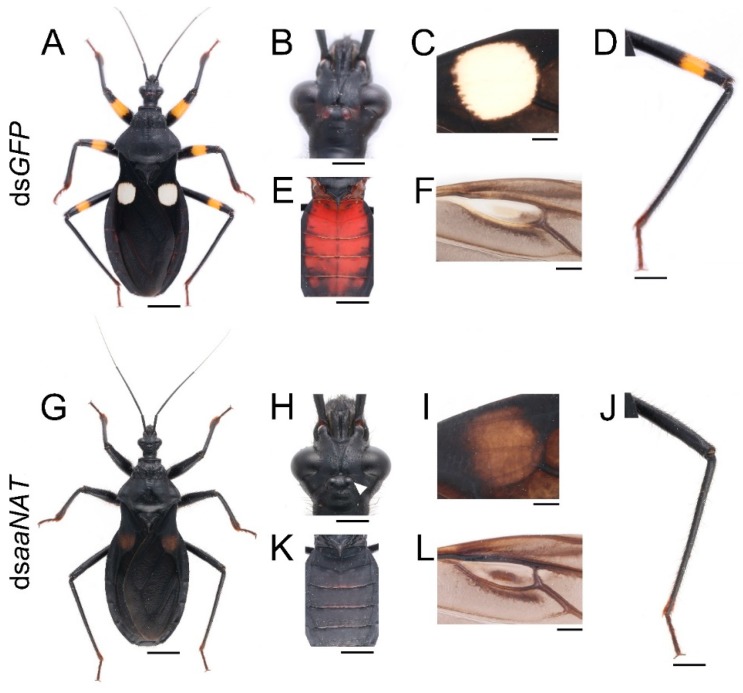
Functions of *aaNAT* in *P. biguttatus* adults. Adults treated with ds*GFP* showed normal pigmentation patterns (**A**), including the brownish red of ocelli (**B**), the white of the hemelytra (**C**), the yellow of the annulation on the leg (**D**), the red of the dorsal abdomen (**E**), and the white of the fenestrate area on the hindwing (**F**). (**G**) Adults treated with ds*aaNAT* showed the expansion of black pigment in the head (**H**), the forewing and the hindwing (**I**,**L**), the leg (**J**), and the dorsal abdomen (**K**). The white arrowhead (**H**) indicates the ocelli. Scale bars, 5 mm (**A**,**E**,**G**,**K**), 2.5 mm (**D**,**J**), 1.25 mm (**B**,**H**), 1 mm (**C**,**F**,**I**,**L**).

To investigate whether *aaNAT* was responsible for the formation of the white spot of the hemelytra, we performed *aaNAT* RNAi in the early fourth-instar nymphs. Of the 21 injected fourth-instar nymphs, no nymphs died, three were used to extract RNA, and 18 molted to fifth-instar nymphs. Then, we injected 20 μg ds*aaNAT* into each of the 18 fifth-instar nymphs (about seven days old), four of them were used to extract RNA, seven molted to adults, and seven died due to aborted ecdysis. Interestingly, RNAi against the *aaNAT* gene generated a distinct pigmentation pattern. The body colors of the fifth-instar nymphs treated with ds*aaNAT* were darker than that of those treated with ds*GFP* (Figure 4G–I,K,L). Moreover, the yellow annulation of the leg turned black after *aaNAT* RNAi (Figure 4G,H,L). However, the color of the sternum was not apparently changed (Figure 4J). A global black phenotype was observed in all adults treated with ds*aaNAT*. The expansion of black pigment was observed throughout the entire body regions, including the brownish red ocelli on the head (Figure 5H, the white arrowhead), the white spot on the hemelytra (Figure 5I), the yellow annulation on the leg (Figure 5J), the red dorsal abdomen (Figure 5K), and the white fenestrate area on the hindwing (Figure 5L). RNAi for *aaNAT* had no effect on the development of cuticles in nymphs or adults (Figure 4 and Figure 5).

## 3. Discussion

### 3.1. Black Pigment Is Solely Generated by the Melanin Pathway

In this study, we report the first application of RNAi in this assassin bug. RNAi has been proven to be an essential tool for studying the functions of genes in non-model insects, for which genetic tools are lacking [32]. However, the efficiency of RNAi varies greatly among different insect species [33]. Among hemipteran insects, RNAi performed poorly in the pea aphid, *Acyrthosiphon pisum*, while it was highly effective in the milkweed bug, *Oncopeltus fasciatus* [32,34]. In this study, the target gene expression level decreased significantly following dsRNA injection (Figure S4). In addition, the phenotype was consistent in the individuals of each RNAi group and could persist until the nymphs died or molted to the next instars. These results make RNAi an effective approach for studying the functions of these three melanin genes in this assassin bug.

The pigmentation process is initiated by *TH*, which hydroxylates tyrosine to DOPA. Our results showed that knockdown of *TH* caused a complete loss of black pigment, with reduced cuticle sclerotization of the compound eyes, the legs, and the wing discs (Figure 2E–H). These results are consistent with those found in *Tribolium*, where downregulation of *TH* led to decreased cuticle hardness and the loss of brown pigment [16]. These results confirm that pigments not only provide visual effects, but also serve as structural components of the cuticle [35,36]. Some dark brown markings were also observed in the ds*TH* nymphs (Figure 2H). A similar phenotype has been reported in *Tribolium* [16]. This may be explained as follows: Due to the loss of *TH* function, tyrosine is excessively accumulated and then converted to the melanin precursor in the cuticle, and finally melanin is synthesized. Results of quantitative real-time PCR showed that *TH* expression level was significantly lower in the first-instar nymphs than in others (Figure 1A). This observation may be explained by the fact that the first-instar nymph has a lower degree of cuticular sclerotization and less black pigment on its body compared with the other instars. Similarly, the level of *TH* expression correlated strongly with the intensity of the black markings in *Papilio xuthus* [19]. The expression level of *TH* was very high just after molting and then gradually decreased during the pigmentation process (Figure 1B). This indicates that *TH* functions mainly at the beginning of the pigmentation. In addition, the *TH* expression level in adult body regions with black and white colors was much higher than that of the nymphs (Figure 1C). These results are consistent with previous studies in *Tribolium* and *Papilio* [16,19]. Taken together, our findings suggest that the black pigment in *Platymeris* is solely generated from the melanin pathway. *TH* functions not only in sclerotization, but also in the formation and the degree of pigmentation. It is noteworthy that downregulation of *TH* did not affect the formation of yellow pigment (Figure 2E). A similar phenotype was found in *O. fasciatus*, in which the orange color persisted in the individuals subjected to *TH* RNAi [29]. This finding suggests that the yellow pigment in hemimetabolous insects is not generated by the melanin pathway, but is synthesized by other pigmentation pathways.

### 3.2. Brown Pigment Could Be Further Converted to Black Melanin

After tyrosine is hydroxylated to produce DOPA, DOPA is then decarboxylated to form dopamine, which can be converted to NADA and NBAD. These substrates are secreted into the cuticle to form different colors. It is a controversial issue whether DOPA or dopamine is the main precursor of black melanin. Previous studies in *Drosophila* indicated that dopamine melanin was the main component of black pigment [7,37]. However, some other analyses provided evidence suggesting that DOPA melanin can contribute equally to black melanin [3,5,6,8,9,38]. In our study, downregulation of *yellow* led to the disappearance of black pigment, while the brown pigment was retained (Figure 3E). The freshly molted assassin bug was bright red, became brown gradually, and then the body turned black after the pigmentation progress was completed (Figures S1 and S2). Furthermore, *yellow* was highly expressed at the brown (five hours after molting) and dark brown stages (ten hours after molting), rather than at the very beginning of the pigmentation progress (Figure 1E). In addition, *yellow* RNAi also caused the brown phenotype in *Oncopeltus* [30]. Taken together, our results demonstrate that dopamine melanin makes a major contribution to black pigment in this assassin bug; however, it is firstly converted to brown pigment, and then the brown pigment is further converted to the black pigment.

In insects, *yellow* is a rapidly evolving gene family, which generates functionally diverse paralogs. In *Drosophila*, *yellow-y* was required to produce black pigment [8,9,39]. A similar function of *yellow-y* was reported in the lepidopteran insects, *P. xuthus* [40] and *B. mori* [41]. In *Tribolium*, *yellow-y* RNAi caused the loss of the pterostigma on the hindwing, whereas no effect was found on pigmentation of the body wall or elytron [14]. *yellow-e* was first studied in *B. mori*, indicating its requirement for the normal pattern of larval body color [42]. Unlike *Bombyx*, *yellow-e* RNAi in *Tribolium* did not affect color formation but caused dehydration-induced mortality in adults [17]. The expression level of *yellow-y* in *Tribolium* was the highest on pupal day three and declined substantially by the time of adult apolysis, while *yellow-e* expression occurred on pupal day three and peaked on pupal day four. In our study, the expression level of *yellow* gradually increased from the first- to fifth-instar nymphs and declined dramatically when the nymphs molted to adults (Figure 1D). This indicates that *yellow* may be essential for normal development in this species. RNAi of *yellow* indeed resulted in high mortality caused by cuticle degradation. Our results indicate that the *yellow* gene in this assassin bug seems to combine the functions of *yellow-y* and *yellow-e*. This *yellow* gene plays an essential role in both pigmentation and cuticle formation.

### 3.3. aaNAT Suppresses the Formation of Black Melanin to Generate Colorless Sclerotin

Finally, we analyzed the NADA branch, which suppresses melanin formation. *aaNAT* functions in this branch, where it can transform dopamine to NADA, after which the colorless sclerotin can be generated [5,6,7,43,44]. Early findings suggested that *aaNAT* was mainly responsible for sclerotization [45,46,47]. It was first demonstrated that *aaNAT* was essential for insect color patterns in *Bombyx* [25,26]. In *Tribolium*, *aaNAT* was not required for larval, pupal, or pharate adult cuticle pigmentation, whereas *aaNAT* RNAi caused a significantly darker body color in adults [28]. However, *Oncopeltus* seemed to recruit *aaNAT* only in the hindwing, as *aaNAT* RNAi showed no effects on color patterns in the head, thorax, or abdomen [30]. Our *aaNAT* RNAi results were consistent with that of *Bombyx*, with a systemic black phenotype observed from the nymphs to the adults (Figure 4 and Figure 5). We noticed that color of the sternum in the fifth instar nymphs was not apparently changed (Figure 4J), this is because this region has a low degree of sclerotization, while there is no such region in adults. The white spot of the hemelytra and the fenestrate area of the hindwing were invaded by black pigment in ds*aaNAT*-treated individuals (Figure 5C,F). The expression level of *aaNAT* was highest in adults, especially in the white spot of the hemelytra (Figure 1G,I). Furthermore, *yellow* was also highly expressed in the white spot (Figure 1F). These results support the “eraser” model proposed by Liu et al. [30]. This model proposes that in the fully black background, *aaNAT* is used for generating the colorless patches by “erasing” the preliminary melanin. Interestingly, *aaNAT* RNAi in this assassin bug led not only to the loss of white pigment, but also to the disappearance of yellow and red color patterns (Figure 5G–L). To investigate whether this phenotype was related to changes in the expression levels of *TH* and *yellow*, we performed quantitative real-time PCR with the *aaNAT* RNAi groups. We found that the expression level of *TH* was not significantly different between ds*aaNAT*-treated nymphs and ds*GFP*-treated nymphs (Figure S5A). However, the expression level of *yellow* was significantly higher in the *aaNAT* RNAi group than that in the *GFP* RNAi group (Figure S5B). We speculate that *aaNAT* could inhibit the expression of *yellow*. When *aaNAT* is downregulated, the high expression of *yellow* can convert the accumulated dopamine to excessive black pigment, and this may be the reason for the global black phenotype in *aaNAT* RNAi groups.

### 3.4. A Modified Melanin Pathway Suitable for P. biguttatus

**Figure 6:**
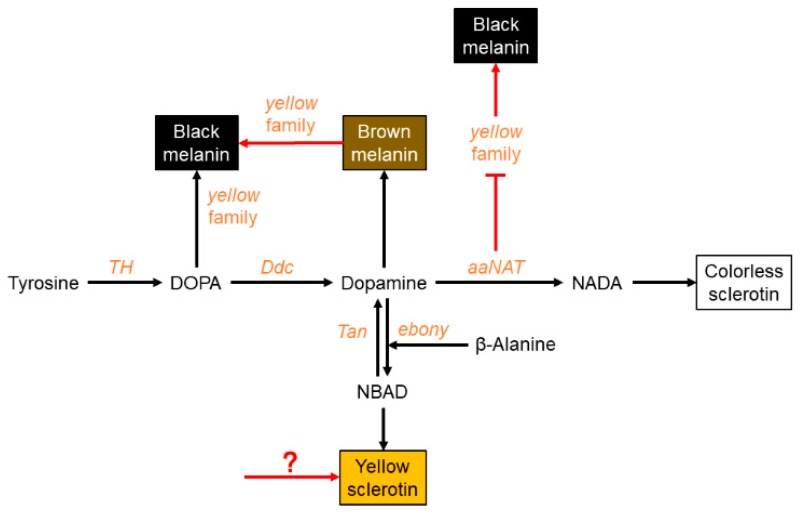
A proposed melanin pathway in *P. biguttatus*. In this assassin bug, black melanin is solely generated from the melanin pathway, and dopamine melanin is the main precursor of black pigment. However, *yellow* functions in the process of converting brown melanin to black melanin (red arrow). The yellow pigment of this species is not synthesized by the NBAD branch, but by other pigmentation pathways. In the NADA branch, *aaNAT* acts as an “eraser” to maintain the white spot on the preliminary black background. Furthermore, *aaNAT* can inhibit *yellow* expression, thereby inhibiting the formation of black melanin (red suppression symbol).

In summary, we provide evidence of a modified melanin pathway that is *P. biguttatus* specific (Figure 6). In this assassin bug, black melanin is solely generated by the melanin pathway. We propose that dopamine melanin is the main precursor of black pigment; however, *yellow* functions in the process of converting brown pigment to black pigment. The yellow pigment of this species is not synthesized by the melanin pathway, but by other pigmentation pathways. In the NADA branch, *aaNAT* acts as an “eraser” to maintain the white sclerotin on the preliminary black background. Furthermore, *aaNAT* can universally suppress the formation of black melanin by inhibiting *yellow* expression.

In this study, we showed that this assassin bug is an excellent model for studying insect pigmentation. We also demonstrated a fascinating phenotype of *aaNAT*, which is completely different from that of *Oncopeltus*. However, it remains unclear why gene functions in this species are more similar to that of holometabolous insects (such as *Tribolium* and *Bombyx*), rather than hemimetabolous insects (such as *Oncopeltus*).

## 4. Materials and Methods

### 4.1. Insects

*P. biguttatus* was reared in the lab for five years. These insects were fed once a week with two species of live larvae: Mealworms, *Tenebrio molitor*, and greater wax moth, *Galleria mellonella*. They were reared under a photoperiod of 16 h/8 h light/dark, at 27 °C and 60% relative humidity during the daytime and at 25 °C and 65% relative humidity at night.

### 4.2. Total RNA Extraction and cDNA Synthesis

The yellow or black regions of the legs were dissected from one fourth-instar nymph, one fifth-instar nymph, and one adult, respectively. The white or black regions of the hemelytra were dissected from five adults. Total RNA of other groups was extracted from one whole insect, except for the first-instar nymph (*n* = 2). Total RNA of each group with three biological replicates was extracted using the RNeasy Mini Kit (Qiagen, Düsseldorf, Germany) following the manufacturer’s instructions. Concentrations of total RNA were measured using a NanoDrop 2000 (Thermo Fisher Scientific, Waltham, MA, USA), and the ratio of A260/A280 (absorbance at 260 nm/absorbance at 280 nm) for all RNA samples was between 1.8 and 2.0. RNA integrity was determined using agarose gel electrophoresis. RNA was considered intact by the presence of a clear 18S rRNA band [48]. All RNA was then treated with DNase I using the DNase Max Kit (Qiagen, Düsseldorf, Germany) following the manufacturer’s instructions. Complementary DNA (cDNA) was synthesized from a standard amount of total RNA (1.9 μg) using the GoScript Reverse Transcription System (Promega, Madison, WI, USA). RNA was incubated with Oligo (dT)_15_ Primer (0.5 μg) and Random Primers (0.5 μg) at 70 °C for 5 min. Each reaction included GoScript Reverse Transcriptase, Recombinant RNasin Ribonuclease Inhibitor, and MgCl_2_. Then incubated at 25 °C for 5 min (anneal), 42 °C for 1 h (extend). Before proceeding with qPCR, inactivated the reverse transcriptase at 70 °C for 15 min.

Sequences of *TH*, *yellow*, and *aaNAT* were identified from the transcriptome data of this assassin bug by performing BLAST searches (*tblastn*) using BioEdit 7.2.5 software (Ibis Biosciences, Carlsbad, CA, USA). These sequences were deposited in the GenBank: MK426755 (*TH*), MK426756 (*yellow*), and MK426757 (*aaNAT*). The sequences of these three genes are given in Table S1, and their orthologs were confirmed by molecular phylogenetic analysis (Figure S3).

### 4.3. RNA Interference

dsRNAs targeting the conserved regions of *TH*, *yellow*, and *aaNAT* were synthesized using the T7 RiboMAX Express RNAi System Kit (Promega, Madison, WI, USA) from the corresponding T7-DNA. T7-DNA was synthesized using pairs of primers containing T7 promoter sequences (TAA TAC GAC TCA CTA TAG G) at the 5′ ends [28]. For the primers used to amplify these target regions, see Table S2. The lengths of the dsRNA products were 483 bp for *TH* (nucleotides 236–718), 448 bp for *yellow* (nucleotides 461–908), and 447 bp for *aaNAT* (nucleotides 118–564). dsRNA for green fluorescent protein (*GFP*) (GenBank number XM_013480425.1, nucleotides 157–576) was used as a control. Primer set for ds*GFP* was 5′-(T7)-CACAAGTTCAGCGTGTCCG-3′ and 5′-(T7)-GTTCACCTTGATGCCGTTC-3′.

The prepared dsRNA was then injected into the abdomen of the early fourth-instar nymphs, using a Hamilton syringe with a 33-gauge needle [49]. A total of 20 μg (approximately 5 μL) dsRNA was injected into each individual. An equivalent amount of ds*GFP* was injected into the control nymphs at the same developmental stage.

### 4.4. Quantitative Real-Time PCR

To determine the efficiency of the RNAi approach and the expression profiles of *TH*, *yellow*, and *aaNAT*, we performed quantitative real-time PCR analyses. The cDNA templates were prepared from total RNA isolated from whole nymphs at 48 h post injection (*n* = 3). Total RNA was independently extracted for each of the three replicates. The sequences of the gene-specific quantitative real-time PCR primers are presented in Table S2. Quantitative real-time PCR was conducted using TB Green Premix Ex Taq II (Tli RNaseH Plus) (Takara, Shiga, Japan) and the CFX96 Real-Time PCR Detection System (Bio-Rad, Portland, ME, USA). The PCR mixtures were contained in the Axygen 0.2 mL thin-wall 8 strip PCR tubes (Corning, Corning, NY, USA), included 2 μL of the synthesized cDNA, 0.5 μL of each primer (10 μM), 12.5 μL of TB Green Premix Ex Taq II (Tli RNaseH Plus), and 9.5 μL of ddH_2_O. Each experiment was performed with three technical replicates and biological replicates. The thermocycling program was as followed: Initial denaturation at 95 °C for 30 s, 40 cycles of 95 °C for 5 s and 60 °C for 30 s, followed by 95 °C for 10 s, and from 65 to 95 °C in increments of 0.5 °C for 5 s [28]. The *elongation factor 1 alpha* (*EF1α*) (GenBank number MK744598) was used as a reference gene. The primers for quantification of *EF1α* expression were 5′-CATCTTTGTTGAGTTTGTCG-3′ and 5′-GACCTGTAGTTGTAGATTTACC-3′. At the end of each quantitative real-time PCR run, a melting curve was generated to confirm the presence of a single peak and rule out the possibility of primer dimer and non-specific product formation. The quantification cycle (Cq) was determined and was used for comparative quantitative analysis. The 2^−ΔΔCt^ method was used to calculate the relative expression levels of the target genes in all groups [50]. Primer amplification efficiencies, correlation coefficient (*r*^2^), slope, and *y*-intercept were presented in Table S3. No template controls (NTC) were performed with every experiment to determine if PCR mixtures were contaminated or unintended products were amplified, such as the primer dimers [51,52]. No Cqs were detected in all NTC experiments. The column chart was drawn using GraphPad Prism 7.0 software (GraphPad, San Diego, CA, USA). SPSS Statistics 25.0 software (IBM, Armonk, NY, USA) was used to perform unpaired Student’s *t* test (for two groups) and one-way ANOVA analysis (for more than two groups).

### 4.5. Image Processing

Images were taken using an SZX7 stereoscope (Olympus, Tokyo, Japan) and a 7D camera (Canon, Tokyo, Japan). The images under a particular magnification were taken under the same light, exposure time, aperture, and white balance conditions.

## Supplementary Materials

The following are available online at https://www.mdpi.com/1422-0067/20/11/2728/s1. Figure S1. Pigmentation process of the fifth-instar nymph. (A) The newly molted fifth-instar nymph was bright red, with a yellow annulation on the leg; (B) over time, the red regions gradually turned brown. (C) The fifth/instar nymph was completely pigmented after about 18 h. (D) The abdomen changed from black to brownish red after feeding. V, fifth/instar nymph; h, hours after molting; AF, after feeding. Scale bar, 5 mm. Figure S2. Pigmentation process of the adult. Similar to the fifth/instar nymph, the future black regions of the adult (D) were first bright red (A), then turned brown (B), and then turned dark brown (C). The adult was completely pigmented after about 48 h. The white spot of the hemelytra remained colorless during the pigmentation process. The region of the dorsal abdomen covered by the forewings was red (E). The membranous hindwing was light brown with a fenestrate area (F, the black arrowhead). A, adult; h, hours after molting. Scale bars, 5 mm (D), 2.5 mm (E,F). Figure S3. Phylogenetic analysis of *TH*, *yellow*, and *aaNAT* based on their deduced amino acid sequences. The molecular phylogenetic tree was constructed by MEGA7 using the neighbor-joining method [53]. Branch support values are bootstrap percentages from 5000 replicates. The scale bars indicate the estimated number of amino acid substitutions per site. Boxes indicate the *P. biguttatus* orthologs. The accession numbers of these sequences are in the parentheses. Figure S4. RNAi efficiency of *TH*, *yellow*, and *aaNAT*. The expression levels of *TH* (A), *yellow* (B), and *aaNAT* (C) were significantly decreased in dsRNA-treated nymphs compared with ds*GFP*-treated nymphs. Data are shown as the mean values ± SE (error bars) (*n* = 3). * *p* < 0.05, ** *p* < 0.01, *** *p* < 0.001, according to the Student’s *t* test. Figure S5. Expression levels of *TH* and *yellow* in ds*aaNAT*-treated nymphs and ds*GFP*-treated nymphs. The expression level of *TH* was not significantly different between ds*aaNAT*-treated nymphs and ds*GFP*-treated nymphs (A). However, the expression level of *yellow* was significantly higher in the *aaNAT* RNAi group than in the *GFP* RNAi group (B). Data are shown as the mean values ± SE (error bars) (*n* = 3). * *p* < 0.05, ** *p* < 0.01, *** *p* < 0.001, according to the Student’s *t* test.

## Abbreviations

*aaNAT*	arylalkylamine-*N*-acetyltransferase
bp(s)	base pair(s)
Cq	quantification cycle
*Ddc*	DOPA decarboxylase
DOPA	dihydroxyphenylalanine
dsRNA	double-stranded RNA
*EF1α*	elongation factor 1 alpha
GFP	green fluorescent protein
NADA	*N*-acetyl dopamine
NBAD	*N*-β-alanyl dopamine
NTC	no template controls
qPCR	quantitative real-time PCR
RNAi	RNA interference
*TH*	tyrosine hydroxylase
