# Supplementary material for: Three Melanin Pathway Genes, TH, yellow, and aaNAT, Regulate Pigmentation in the Twin-Spotted Assassin Bug, Platymeris biguttatus (Linnaeus)
Source: Int J Mol Sci. 2019 Jun 3;20(11):2728. doi: 10.3390/ijms20112728 (PMC6600426; doi:10.3390/ijms20112728)
Supplement: Supplementary file 1 [file ijms-20-02728-s001.zip › Supplementary Files/Table S1.docx]

>*TH*

ATGATGGCCGTAGCTGCAGCTCAGAAGAACAGGGAGATGTTCGCCATTAAAAAATCTTACAGCATCGAGAATGGGTACCCAGCAAGAAGAAGATCTTTAGTAGATGATGCACGTTTTGAAAGTGTTGTCGTTAAACAAACTAAACAGAGTGTACTCGAGGAAGCAAGGATTAAACATAATGACAATGCTGAGGTAAATGCAAATCAAACTAATTTGAAAGAAGATGTCAAAAAATCATCTGACATTAGGGAACCTCATGAGCAAAAATTTACAGTTGAAATAGATGATGAAAACAAAGAAAATGTTTGCGATGATCAACCACAAATGCAAAATGGACATACAGATGCTGGTTTAACTGAAGAAGAAGTTATCCTAAGTAATGCAGCCAGTGAAAGTAAAGAAGCTGAACAAGCTATACAAAGAGCCGCTTTAATTCTAAAATTAAAGGAAGGAATGGGATCATTGGCTCGCACTTTGAAATCTATTGAGAACTTTAAAGGAACAATAGTTCATTTGGAAACAAGACCTTCCAAGGATGCTGGTGTACAATTTGACGTACTAGTTAAAGTTGATATGTCACGCCAAAACTTATTGCTTCTCATTAGATCTCTTAGGCAAAGCGCTTCTTTGGGTGGAGTTGATCTCCTAGCCGACAATAAGATTAGCCTAAAAAATCCGTGGTTCCCACGTCATGCCCGTGACTTGGATAACTGTAACCACTTAATGACGAAATATGAACCTGAACTTGATATGGCTCACCCAGGATTCTCTGACCAGGTGTACAGAGAAAGACGTAGAGAAATTGCAAATATCGCTTTTGAATATAAATATGGAGATCCAATCCCATTCATCCAATACACCGATGATGAAAGTGCAACATGGGAAGCTGTCTACAAGACTGTAGTAGAATTGATGCCAAAACATTTCTGCAAAGAATATAAGGTACAATTTGCTAGAATGGAAGCTGAAGGAATTTTCGCACCAAAAACTATACCACAATTAGAAGATGTATCAAATTTCTTGAAGAAAAGTACTGGTTTTACTCTACGTCCTGCTGCAGGATTATTAACATCACGAGATTTTCTTGCCAGTCTTGCATTTAGAGTATTTCAGAGTACTCAATATATTAGACACAATACTACCCCATTTCATACACCAGAACCTGATTGTGTCCATGAACTCTTAGGACATATGCCATTACTTGCTGATCCAGCTTTTGCACAATTTTCACAAGAGATTGGGCTAGCATCATTGGGTGCTAGTGATGAGGAAATTGAAAAATTATCAACAGTATATTGGTTTACTGTTGAATTTGGTATGTGTAAAGAGCATGGTAATTTAAAAGCCTATGGTGCCGGCCTATTATCTAGCTATGGTGAATTATTACATTCAATATCTGATAAACCAGAACATAGACCATTTGAACCAGCTATAACAGCTTTACAACCATACCAGGATCAAGATTATCAACCAATATATTATGTAGCTGAAAGTTTTGAAGATGCTAAAGAGAAATTTAGAAGATGGGTATCAACCATGTCTAGACCATTTGAAGTTAGATATAACCCACACACACAAGAAGTTGAAGTTTTGGATTCAGTTGATAGGTTGGATAATCTAGTATCACATTTGAATTTGGAAATGCAACATCTTACTACAGCTATAAACAAACTGAGAGCGACATTTGGTTAG

>*yellow*

ATGTGGACCTTATACATTATTAATATTTTAGCTTTGACCAGCTATACACATGCAGCAGCTAACCTATATGGAAAATTTGCTTGGAAATCTTTAGATTATGCTTTTCCTTCGGAATATAGCAGAACGGAAGCTTTATACACAGGAGATTTCATACCAGAAAATAATCTACCAGTAGGAATTGAAATATGGAAGAACAAAATGTTTATAACAGTTCCAAGGTGGGATAAAGGTGTACCATCTACCCTAAATTATGTACCTTTGGATATCGCTTACGATGAGTCACCAAAATTGATACCATACCCAAATTGGGAAACTAATAAGGAAGGTAATTGCCAAGGATTGACTACTACTTACAGAGTAAGAGTAGACGAATGTGACAGATTATGGGTACTGGATTCAGGCACAGTTGGAATAGGAAATTTAACTCAACAAGTTTGTCCTTATGCACTTCATGCCTTTAATCTAAAGAATGACCGCCAGATCTTAAGATATCAATTTAAAGATGATGATGTCAATGCAAATACATTTATTGCAAACATTGCAGTAGAAGTTGGTCATACATGCGATGATACATGGGTATATGCTTCTGATGAATTAGGTTATGGTCTACTTGTTTATGATTTAAAAGAAAATGATTCATGGAGATTTGAACATGGTTTCTTCCATCCAGATCCATTAAAAGGTGATTTCAATATAGCAGGACTAAATTTTCAATGGGAAGCTGAAGGAATATTTGGTATAGCTTTGTCACCTAGAAACAGTATAGAACGTTTACTCTTCTTTCATCCATTAGCAAGTCATAGAGAATTTGTAGTACCCACTAATGTACTGAAAGCTAAGCCTGATAAAGATTCTTATCATAAGTTTATAGCATTAGCTGAACATCTAGGACATTCAACAGCACAAACTATGACTGAAGATGGAATAATGCTTTTTAATTTAGTTGATCTTAATGCTATAGGATGTTGGAATTGGAATGATCCATATGATATAAAACATCAAGCTATTGTTGCCAAAGATGATATAGCTCTAGTCTTCCCTAGTGATGTACGTGTTGTTGATGGCAATGTTTGGGTTATGTCAGATCGTATGCCAGTACATTTAATTTCTAAACTTGATTTTAATGACGTTAATTTCAGAGTTATGTTCTTCTCATTACACGAAGCAGTTTCGGGTACTGTTTGTCAAAATGGTAAAACCGGTATACATAATGCCATTTTACCCCTTTAA

>*aaNAT*

ATGGAAACTATTAACAGTGAAATAAAATCATTGTCTTCGCCCTTCATTGTCAATGATAAGAAAGAATTACTAGTACAAAAAGGCAAGATGGAAGACAAAGGCTACAGGATAGTTACCATGACCGAAAGAGACACCAACGATGCCATAATGTTTTTAAGGAAATTTTTTTTTCATGATGAACCATTAAATGTATGTGTTGGATTATTGGATGAACCAGGTTCTACTTGCCAAGAATTGGAAGATTACTGTTCCATTTCAATTCCTGATGGATTATCGTTAAAGGCAGTATCACCTACTGATGAAATAATAGCTGTTTGTATTAATGGATCATTAACTAGGGAAAACAATAAAGTAGACGCAATGATTGAAGAAGTAAACTCTTGCAAAAACCAAAAATTCAAAAAAATATTAAATTTAATTACATCTGTTAATATACAATCAGATATTTTTGGACAGTTCCCAAAAATTAACAGTATGGCTGAAGTTAGAGTATTGTCCGTTGATGATGCATACAGAGGAAAAGGAATTGCTAAAGCATGTATTGAGAGAACCAGGGTATTAGCCAAAGAAAAAGGATATGACCTATTGAAATTGGACTGTACTAGTCATTATTCAGCGTTAGCTGTATCAAGCCTTGGAGGATACAGTTGTGTTTATACGCTCAATTATTCTGATCATGTAGATGAAGATGGAAAGCCAGTATTTGTACCAGAACTACCGCATTCATGTGTAAAAACTTTTGTATGCAGTTTAAAATAA
