# Supplementary material for: Three Melanin Pathway Genes, TH, yellow, and aaNAT, Regulate Pigmentation in the Twin-Spotted Assassin Bug, Platymeris biguttatus (Linnaeus)
Source: Int J Mol Sci. 2019 Jun 3;20(11):2728. doi: 10.3390/ijms20112728 (PMC6600426; doi:10.3390/ijms20112728)
Supplement: Supplementary file 1 [file ijms-20-02728-s001.zip › Supplementary Files/Table S2.docx]

| Target | Primers for dsRNA production | Primers for quantitative real-time PCR |
| --- | --- | --- |
| *TH* | Forward: 5'-(T7)-CATCTGACATTAGGGAAC-3' | Forward: 5'-GGCTCGCACTTTGAAATC-3' |
|  | Reverse: 5'-(T7)-GGTTACAGTTATCCAAGTC-3' | Reverse: 5'-GGCTAATCTTATTGTCGGC-3' |
| *yellow* | Forward: 5'-(T7)-ATCTAAAGAATGACCGCCAG-3' | Forward: 5'-CAAGGTGGGATAAAGGTGT-3' |
|  | Reverse: 5'-(T7)-GTTTGTGCTGTTGAATGTCC-3' | Reverse: 5'-CCTATTCCAACTGTGCCTG-3' |
| *aaNAT* | Forward: 5'-(T7)-ATGACCGAAAGAGACACC-3' | Forward: 5'-TAGAGTATTGTCCGTTGATG-3' |
|  | Reverse: 5'-(T7)-GGCTAATACCCTGGTTCT-3' | Reverse: 5'-GCTAACGCTGAATAATGAC-3' |

**Table S2. Primers used for dsRNA production and quantitative real-time PCR.**
