# Supplementary material for: Three Melanin Pathway Genes, TH, yellow, and aaNAT, Regulate Pigmentation in the Twin-Spotted Assassin Bug, Platymeris biguttatus (Linnaeus)
Source: Int J Mol Sci. 2019 Jun 3;20(11):2728. doi: 10.3390/ijms20112728 (PMC6600426; doi:10.3390/ijms20112728)
Supplement: Supplementary file 1 [file ijms-20-02728-s001.zip › Supplementary Files/Table S3.docx]

**Table S3. Primer information obtained from the standard curves.**

| Gene | Amplification efficiency | *r^2^* | slope | *y*-intercept |
| --- | --- | --- | --- | --- |
| *TH* | 84.9% | 0.978 | 3.745 | 30.499 |
| *yellow* | 96.3% | 0.990 | 3.414 | 33.132 |
| *aaNAT* | 91.3% | 0.973 | 3.551 | 35.782 |
| *EF1α* | 85.5% | 0.980 | 3.726 | 28.093 |
