# Supplementary figures and images for: Three Melanin Pathway Genes, TH, yellow, and aaNAT, Regulate Pigmentation in the Twin-Spotted Assassin Bug, Platymeris biguttatus (Linnaeus)
Source: Int J Mol Sci. 2019 Jun 3;20(11):2728. doi: 10.3390/ijms20112728 (PMC6600426; doi:10.3390/ijms20112728)

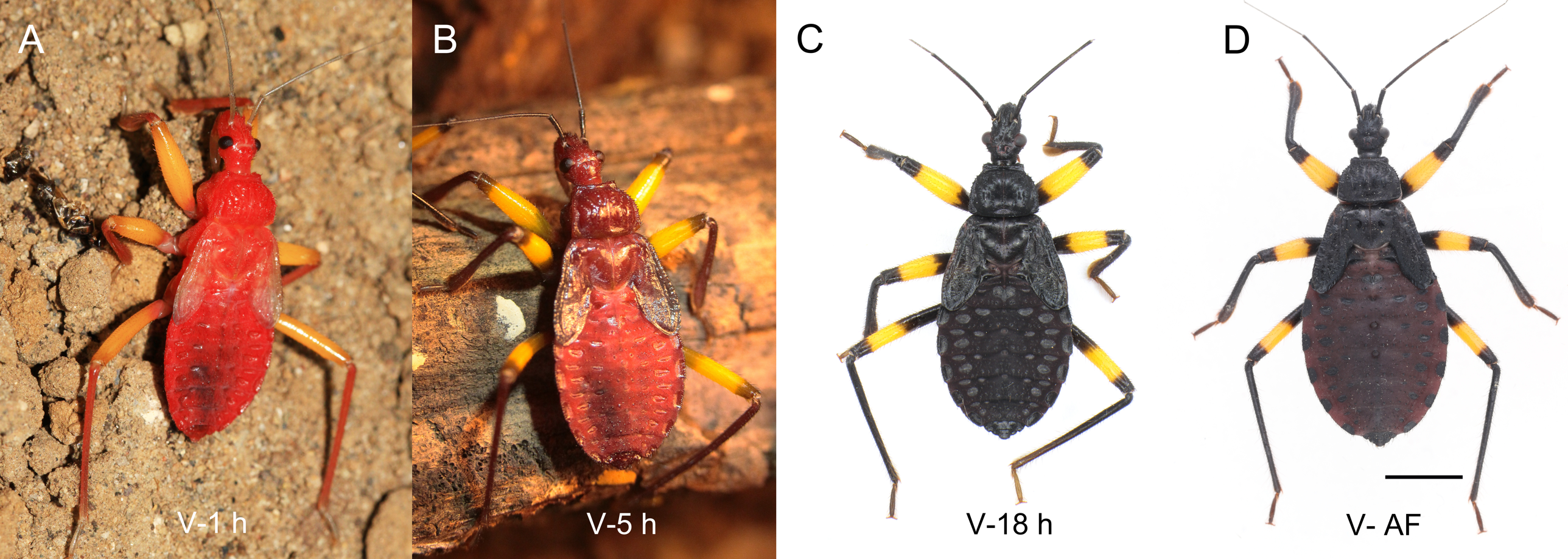

Supplement: Supplementary file 1 [file ijms-20-02728-s001.zip › Supplementary Files/Supplementary Figure S1.jpg]

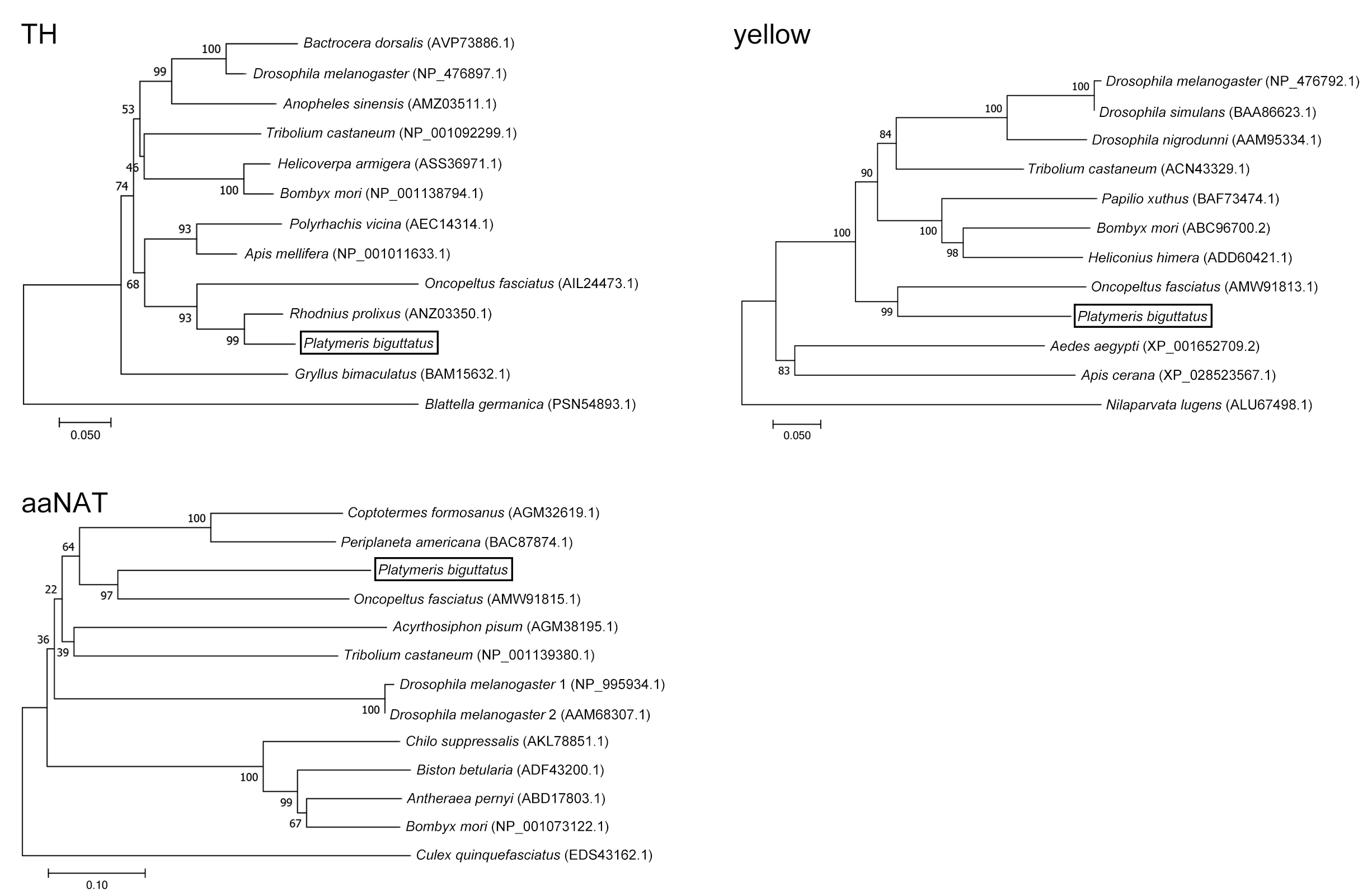

Supplement: Supplementary file 1 [file ijms-20-02728-s001.zip › Supplementary Files/Supplementary Figure S3.tif]

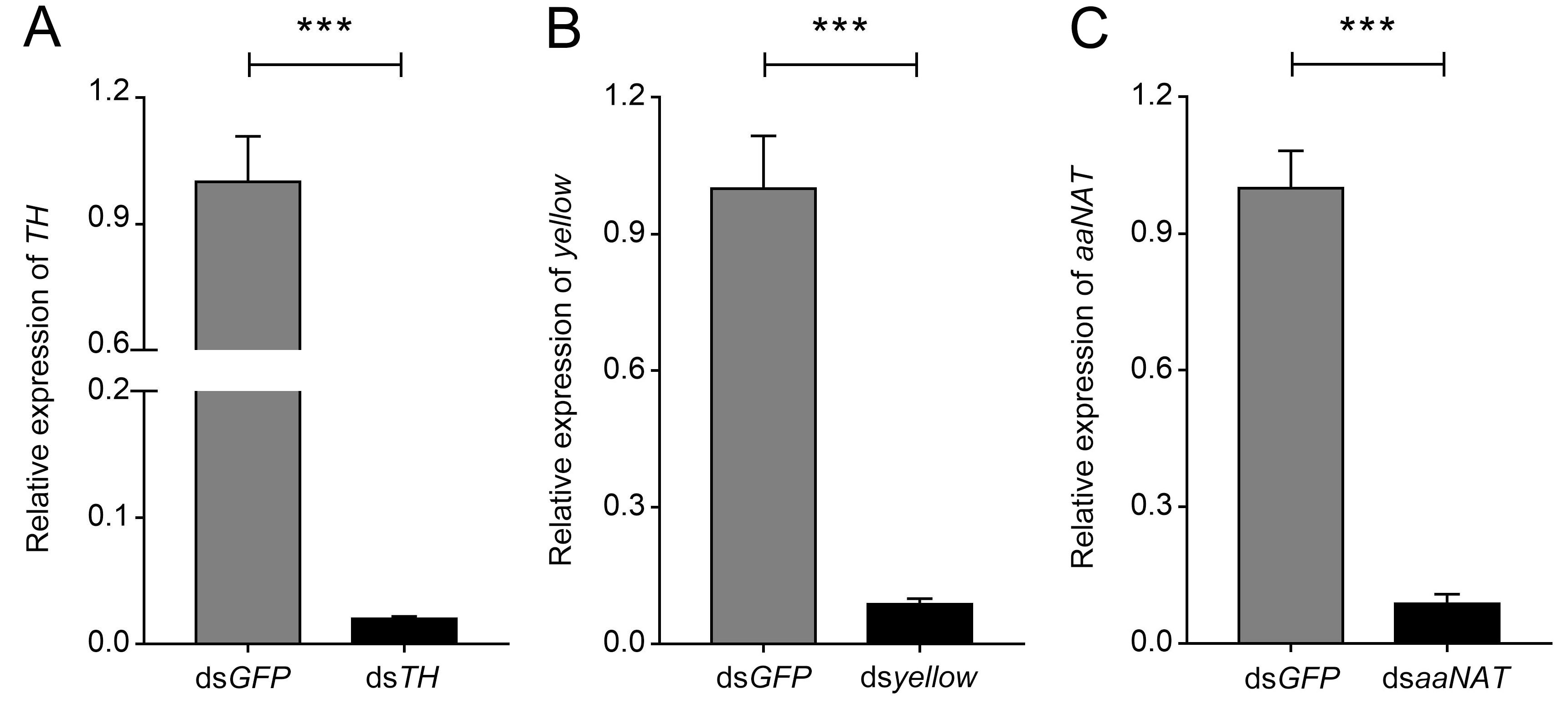

Supplement: Supplementary file 1 [file ijms-20-02728-s001.zip › Supplementary Files/Supplementary Figure S4.jpg]

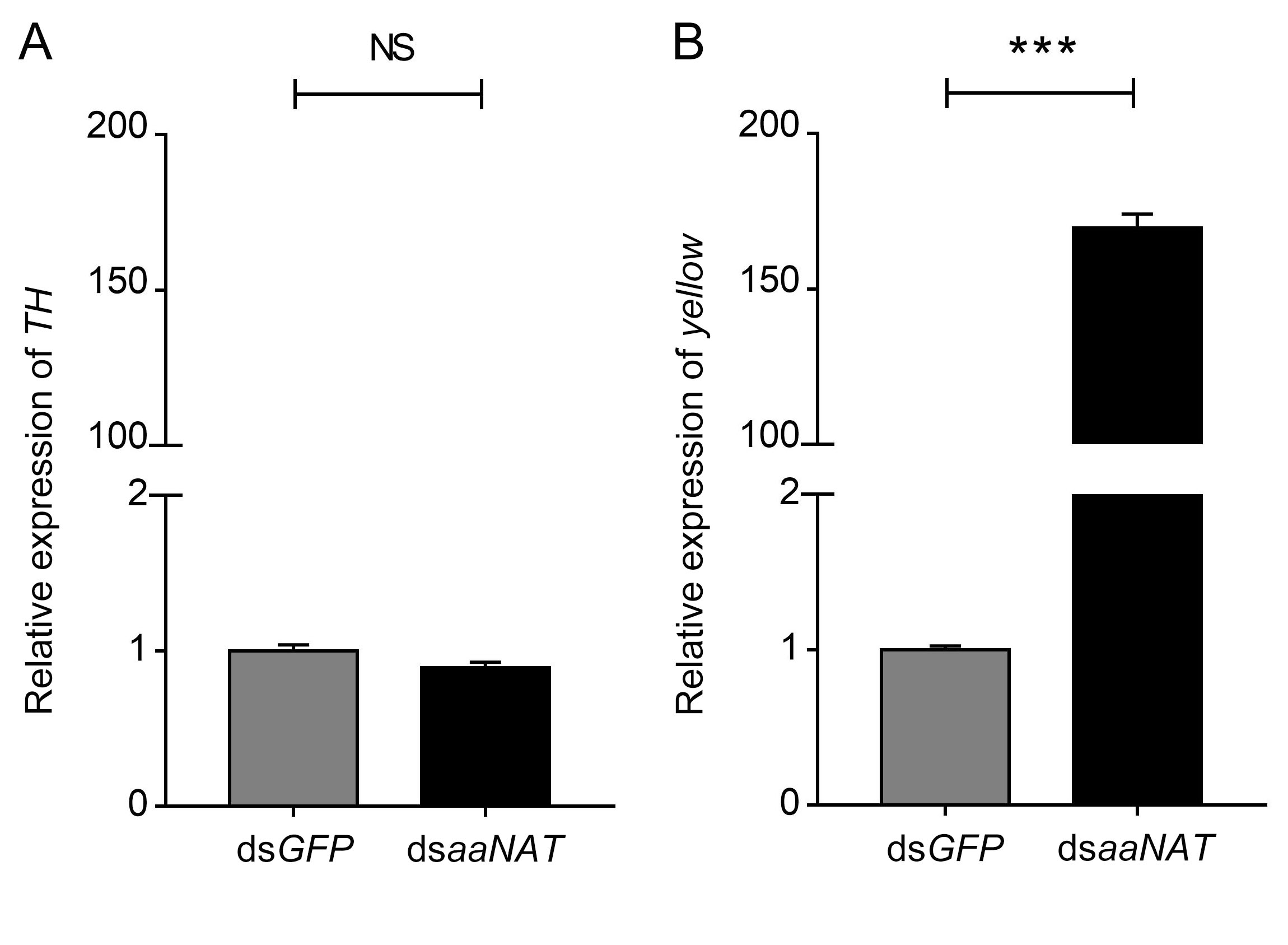

Supplement: Supplementary file 1 [file ijms-20-02728-s001.zip › Supplementary Files/Supplementary Figure S5.jpg]
